# The Clinicopathological Distinction Between Seropositive and Seronegative Immune-Mediated Necrotizing Myopathy in China

**DOI:** 10.3389/fneur.2021.670784

**Published:** 2021-07-05

**Authors:** Xue Ma, Li Xu, Suqiong Ji, Yue Li, Bitao Bu

**Affiliations:** Department of Neurology, Tongji Medical College, Tongji Hospital, Huazhong University of Science and Technology, Wuhan, China

**Keywords:** immune-mediated necrotizing myopathy, seronegative, anti-signal recognition particle antibodies, anti-3-hydroxy-3-methylglutarylcoenzyme-a reductase antibodies, myalgia, membrane attack complex, subclinical cardiac involvement

## Abstract

**Objectives:** The present study aimed to compare the clinicopathological features of patients with seronegative immune-mediated necrotizing myopathy (IMNM) and those positive for anti-signal recognition particle (SRP) or anti-3-hydroxy-3-methylglutarylcoenzyme-a reductase (HMGCR) antibodies.

**Methods:** We retrospectively analyzed the data of patients with IMNM treated in the Neurology Department of Tongji Hospital from January 1, 2013, to December 31, 2019.

**Results:** Among the 117 patients with IMNM, 30.8% (36/117) were positive for anti-SRP antibodies, 6.0% (7/117) were positive for anti-HMGCR antibodies, and 13.7% (16/117) were seronegative. Myalgia at presentation (62.5 vs. 23.3%, *p* = 0.0114) was more commonly observed in patients with seronegative IMNM than in those with seropositive IMNM. Subclinical cardiac involvement was more frequently detected in seronegative IMNM than in seropositive IMNM (6/13 vs. 5/33, *p* = 0.0509, echocardiogram; 7/7 vs. 12/24, *p* = 0.0261, cardiac MRI). Deposition of membrane attack complex (MAC) on the sarcolemma of myofibers in biopsied muscle was less commonly observed in patients with seronegative IMNM than in patients with seropositive IMNM (16.7 vs. 68.2%, *p* = 0.0104). The rate of marked improvement following immunotherapy tended to be higher in patients with seronegative IMNM than in those with seropositive IMNM (87.5 vs. 61%, *p* = 0.0641).

**Conclusions:** Patients with seronegative IMNM more frequently present with myalgia at onset, exhibit more subclinical cardiac involvement and uncommon MAC deposition on myofibers, and experience better outcomes than those with seropositive IMNM.

## Introduction

Immune-mediated necrotizing myopathy (IMNM) is a relatively novel clinical entity among autoimmune myopathies and is clinically characterized by subacute muscle weakness in the proximal limbs and trunk in combination with elevated levels of serum creatine kinase (CK) (1). Skeletal muscle biopsies obtained from patients with IMNM generally exhibit prominent myofiber necrosis and regeneration, as well as a scarcity of inflammatory infiltrates (2–5).

Several subtypes of IMNM have been identified, including myositis autoantibody-associated IMNM (6, 7), connective tissue disease (CTD)-related IMNM (4, 8), statin-related IMNM (9–12), cancer-related IMNM (13–15), immune checkpoint inhibitor-induced IMNM (16), and IMNM related to unknown causes. Myositis antibodies are considered a cornerstone in the study of immunopathological mechanisms and diagnostic markers, including myositis-specific antibodies (MSAs) and myositis-associated antibodies (MAAs) (17). MSAs are typically divided into several subgroups. The IMNM-specific antibodies include anti-signal recognition particle (SRP) and anti-3-hydroxy-3-methylglutaryl-coenzyme a reductase (HMGCR) antibodies (6, 7). Anti-aminoacyl-tRNA synthetase (ARS) antibodies usually consist of anti-histidyl-tRNA synthetase (Jo-1), anti-alanyl-tRNA synthetase (PL-12), anti-glycyl-tRNA synthetase (EJ), anti-isoleucyl-tRNA synthetase (OJ), and anti-threonyl-tRNA synthetase (PL-7) autoantibodies. Dermatomyositis (DM)-associated antibodies routinely include anti-mitochondrial (Mi) 2α and β, anti-transcriptional intermediary factor 1γ (TIF1γ), anti-melanoma differentiation-associated protein 5 (MDA5), anti-nuclear matrix protein 2 (NXP2), and anti-small ubiquitin-like modifier activating enzyme 1 (SAE1) antibodies. The inclusion body myositis (IBM)-specific antibodies are anti-cN-1A antibodies (18). MAAs include anti-Ku, anti-Ro52, anti-polymyositis-scleroderma 100 protein (PMScl100), anti-polymyositis-scleroderma 75 protein (PMScl75), anti-ribonucleoprotein (RNP), and anti-mitochondrial antibodies, which can be detected in autoimmune myopathies as well as connective tissue diseases (19, 20). In patients with autoimmune myopathies, different myositis antibodies are usually associated with variations in clinical manifestations, muscle pathology, and prognosis (21). MSA-negative IMNM is characterized by frequent occurrence of extra muscular disease activity (EMA), female predominance (8), and a higher risk of tumors (13).

We previously reported that a case with seronegative IMNM suffered a severe recurrence during pregnancy, which likely occurred due to discontinuation of immunotherapy and pregnancy (22). Due to a lack of reliable markers, seronegative IMNM may easily be misdiagnosed or mistreated. In the present study, we aimed to draw attention to seronegative IMNM by retrospectively comparing seronegative IMNM with seropositive IMNM, which included anti-SRP autoantibody-positive or anti-HMGCR autoantibody-positive IMNM.

## Materials and Methods

### Patient Selection

**Figure 1:**
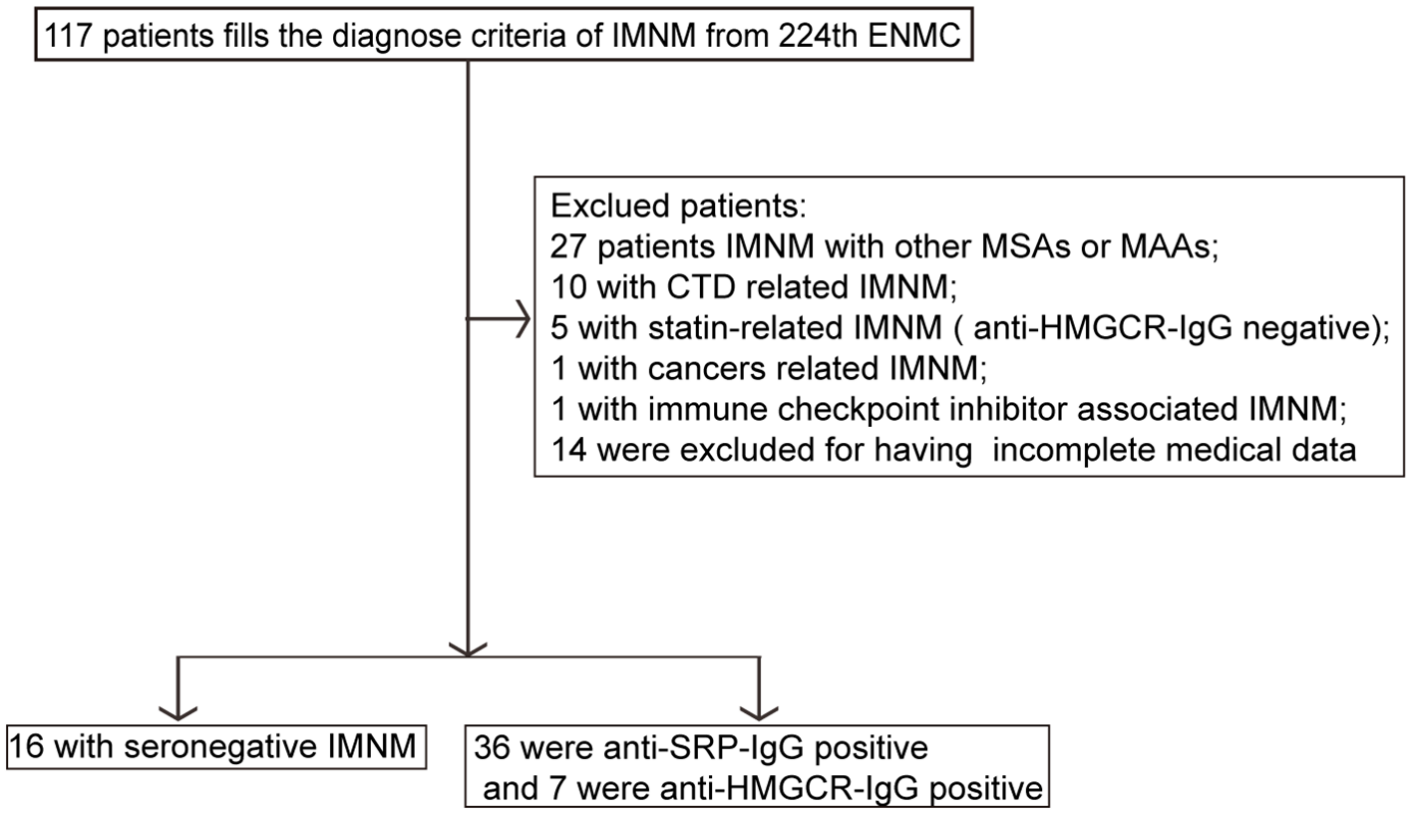
Study flow diagram. CTD, connective tissue disease; ENMC, European Neuromuscular Centre; IMNM, immune-mediated necrotizing myopathy; HMGCR, anti-3-hydroxy-3-methylglutarylcoenzyme a reductase; MAAs, myositis-associated antibodies; MSAs, myositis-specific antibodies; SRP, signal recognition particle; IgG, immunoglobulin G.

We searched the patient database of the Department of Neurology at Tongji Hospital from January 2013 to December 2018 using the following keywords: autoimmune myopathies, idiopathic inflammatory myopathies, IMNM, necrotizing autoimmune myopathy, and polymyositis. Diagnostic criteria for IMNM were based on the inclusion criteria specified by the European Neuromuscular Centre (ENMC) International Workshop on Idiopathic Inflammatory Myopathies (4, 21). Adult patients who predominantly presented with proximal muscle weakness, myofiber necrosis and regeneration, and a scarcity of inflammatory infiltrates were selected. Patients in whom a specific cause of myositis was identified during follow-up were excluded from the study. The selected seronegative patients underwent follow-up for at least 1 year. The diagnostic criteria for seronegative IMNM are shown in Table 1. A total of 59 patients with IMNM (*n* = 117) were enrolled. Among them, 30.8% (36/117) patients were seropositive for anti-SRP antibodies, 6.0% (7/117) were positive for anti-HMGCR antibodies, and the remaining 13.7% (16/117) were seronegative. The exclusion criteria were as follows: IMNM with other MAAs or MSAs (*n* = 27), connective tissue diseases (*n* = 10), statin-related IMNM (*n* = 5), cancer-related myopathy (*n* = 1), and use of immune checkpoint inhibitors-associated IMNM (*n* = 1). No cases of statin-induced anti-HMGCR myopathy existed. The study flow diagram is displayed in Figure 1. The distribution of IMNM cases excluded due to the presence of other MAAs or MSAs is shown in Supplementary Figure 1. The study was approved by the Ethics Committee of Tongji Hospital (IRB ID: TJ-C20121221), and all participants provided written informed consent.

**Table 1:** Diagnostic criteria of seronegative IMNM.

**Criteria**
1. Clinical features
Onset usually after 18 years
Subacute or insidious onset
Pattern of weakness: symmetric proximal>distal, neck flexor>neck extensor
2. Elevated serum CK level
3. No MSAs or MAAs detected in serum
4. Other laboratory criteria (1 of 2):
(a) EMG: myogenic discharges but not the pattern of myotonic dystrophy or other channelopathy
(b) MRI: diffuse or patchy edema within muscle tissue
5. Muscle biopsy
(a) Pronounced necrotic and regenerating muscle fibers
(b) Scarcity of inflammatory cell infiltration
6. Exclude CTD-related, statin-related, cancer-related, and immune check point-induced IMNM

*CK, creatine kinase; CTD, connective tissue disease; EMG, electromyography; IMNM, immune-mediated necrotizing myopathy; MAAs, myositis-associated antibodies; MSAs, myositis-specific antibodies; MRI, magnetic resonance imaging*.

### Clinicopathological Data

Demographic data (age at onset and sex), mode of affected muscles, muscle strength evaluation using manual muscle testing using the Medical Research Council (MRC) scale (23), serum CK and lactate dehydrogenase (LDH) levels, duration of follow-up, presence of EMA, muscle biopsy features, treatment response, and outcomes were documented. EMA included fever, arthritis, Raynaud phenomenon, interstitial lung disease (ILD), and skin symptoms. ILD was detected using chest computed tomography (CT). Electromyography (EMG) and nerve conduction studies were performed.

Manifestations of muscle edema, fatty replacement, atrophy, and myofascial edema were evaluated using muscle magnetic resonance imaging (MRI). Edema, fatty replacement, atrophy, and myofascial edema were defined in accordance with previously described methodology (24). Cardiac abnormalities were assessed *via* electrocardiograms (ECGs), echocardiograms (Echo), and cardiac MRI.

All patients underwent skeletal muscle biopsy for pathological analysis. Serial thick frozen sections (thickness: 7 μm) were stained using routine methods including hematoxylin–eosin, modified Gomori's trichrome, acid phosphatase, NADH-tetrazolium reductase, Sudan black, cytochrome C oxidase, succinate dehydrogenase, periodic acid-Schiff, oil red O, and myosin ATPase. Immunohistochemical (IHC) staining was performed to identify inflammatory cells, including CD68^+^ macrophages (1:50, ab201340, Abcam), CD4^+^ T cells (1:50, ab133616, Abcam), CD8^+^ T cells (1:50, ab93278, Abcam), and CD20^+^ B cells (1:200, PB9050, Boster). The expression of major histocompatibility complex class I (MHC-I) (1:100, ab23755, Abcam) on the sarcolemma, deposition of membrane attack complex (MAC) (1:50, Sc-58935, Santa Cruz Biotechnology) on the sarcolemma and vasculature, and presentation of p62 (sequestosome 1) (1:200, 18420-1-AP, Proteintech) were also analyzed *via* IHC.

Biopsied muscle samples for IHC analysis that did not include more than 300 myofibers were excluded from this analysis. For semi-quantification of CD68^+^ macrophages, CD4^+^ T cells, CD8^+^ T cells, and CD20^+^ B cells, cell counts of 10 high-power fields (HPFs, one HPF as 200×) were analyzed for each biopsy specimen. The average cell count was graded as follows: 0 = almost no staining (<5 positive cells/HPFs); 1 = less staining (5–20 positive cells/HPFs); 2 = more staining (21–50 positive cells/HPFs); and 3 = abundant staining (>50 positive cells/HPFs). For MHC-I, positive staining was regarded as upregulation of the sarcolemma; otherwise, staining was regarded as negative if only endomysial capillaries were stained. Positive MAC and p62 staining were regarded as the presence of at least one myofiber with cytoplasm and at least one sarcolemma or blood vessel with MAC deposition.

### Autoimmune Serologic Testing

All serums from enrolled patients were tested for MSAs, MAAs, and CTD-related factors. The following MSAs and MAAs were assessed using two commercial semi-quantitative line blot assays (D-Tek, Germany; Euroline, Germany): anti-Mi2α and β, anti-TIF1γ, anti-MDA5, anti-NXP2, anti-SAE1, anti-Jo1, anti-SRP, anti-HMGCR, anti-PL7, anti-PL12, anti-EJ, anti-OJ, anti-cN-1A, anti-Ku, anti-PMScl100, anti-PMScl75, and anti-Ro52 antibodies (19). The tests for anti-nuclear, anti-SSA/Ro60, anti-SSB/La, anti-Sm, anti-RNP, anti-mitochondrial, anti-dsDNA antibodies, and rheumatoid factors were performed at Tongji Hospital Laboratory.

### Treatment Outcome Measures

Outcomes were assessed according to the MRC grade of the weakest muscle group: none (MRC grade 5), mild (MRC grade ≥4/5), moderate (MRC grade 3–4/5), and severe (MRC grade <3/5). Outcomes were graded as no improvement, mild improvement (1 MRC grade in 1–2 muscle groups, severe disability, or persistently requiring aids for activities of daily living), moderate improvement (>1 MRC grade in multiple muscle groups, demanding minimal assistance with activities of daily living), and marked improvement (symptoms and signs of mild weakness and normal or near-normal functioning) (3). In addition, the following clinical parameters were evaluated in all patients once every 2–3 months: serum CK levels, LDH, myoglobulin, regular laboratory results (routine blood and urine tests), renal and hepatic function, and blood glucose.

### Statistical Analysis

Descriptive statistics were used to present the results. Categorical variables were reported as frequencies and percentages. The Mann–Whitney *U*-test was used for continuous variables, while a two-sided Fisher's exact test was used to yield proportions for categorical variables. Statistical analysis was performed using GraphPad Prism 8.01 software (GraphPad Software, Inc., La Jolla, CA, USA). Variables with two-tailed *p*-values <0.05 were considered statistically significant.

## Results

### Clinical Features of the 16 Patients With Seronegative Immune-Mediated Necrotizing Myopathy

Detailed clinical information for the enrolled patients with seronegative IMNM is presented in Supplementary Table 1. The median age at onset of the myopathic symptoms was 47 years (range, 24–73 years), and eight patients (50%) were female. No family history of hereditary myopathy or muscular dystrophy was detected in these patients. Limb weakness was evident in 15 patients (95.5%), with proximal muscles preferentially affected. Ten patients (62.5%) reported myalgia. No obvious cardiac symptoms were noted in any of the included patients.

Serum CK levels in the 16 patients ranged from 414 to 20,000 U/L. EMG revealed myogenic changes, such as short-duration and low-amplitude motor units with early recruitment in all patients. EMG also showed fibrillation potentials in four of these patients. MRI abnormalities included muscle edema (80%), fatty replacement (20%), muscle atrophy (26.7%), and myofascial edema (20%), which were mainly distributed in the pelvic muscles and thigh muscles.

The age range of seronegative patients with IMNM with cardiac abnormalities was 33–73 years, and no patient reported a cardiac history. ECG was performed in 14 patients. Arrhythmia was detected in six patients (42.9%), including conduction blockade, aberrant repolarization of the myocardium, tachycardia, changes in T waves, or premature atrial beats. Cardiac ultrasound was performed in 13 patients. Six cases (46%) exhibited systolic dysfunction, aortic stenosis, pericardial effusion, or enlargement of cardiac chambers. Furthermore, cardiac MRI (completed in seven cases) revealed myocardial fibrosis, ventricular or atrial dilatation, or myocardial ischemia in seven patients (100%).

All patients received immunotherapy after providing informed consent. In all cases, initial therapy included high-dose glucocorticoid treatment (methylprednisolone 500 mg/day or 1,000 mg/day intravenously for 3–5 days, followed by tapering oral prednisone). Five patients (5/16) required additional immunosuppressants to achieve remission, including tacrolimus (*n* = 3), mycophenolate mofetil (*n* = 1), and intravenous immunoglobulin (*n* = 1). At the last interview, 14 patients (14/16) had achieved marked improvement. The remaining two patients (2/16) exhibited moderate improvement and could perform activities of daily living with minimal assistance. Of the two patients with moderate improvement, one developed dilated cardiomyopathy and heart failure 1 year later, although immunotherapy had significantly improved the presentation of myopathy and normalized CK and LDH levels. Four patients (4/16) experienced relapses while tapering prednisone, which presented as re-elevated CK levels (1,000–3,000 U/L) and decreases in MCR grades. Subsequently, the relapse resolved after combined treatment with prednisone and mycophenolate mofetil in one patient, tacrolimus in two patients, and azathioprine in one patient.

### Comparison of Clinical Features, Pathological Changes, and Outcomes Between Seropositive and Seronegative Patients

The comparison between seropositive patients, including patients with anti-SRP antibodies and patients with anti-HMGCR antibodies, and seronegative patients is summarized in Table 2. The proportion of patients with myalgia was significantly higher among seronegative patients (62.5 vs. 23.3%, *p* = 0.0114) than among seropositive patients. Overall, there were few differences in demographic characteristics or clinical presentation between seropositive and seronegative IMNM.

**Table 2:** Comparison between patients with seropositive and seronegative IMNM.

**Items**	**All patients (*n* = 59)**	**Seropositive (*n* = 43)**	**Seronegative (*n* = 16)**	***p*-value**
**Demographics**				
Age at onset, median (range), years	48 (9–73)	48 (9–71)	47 (24–73)	0.2563
Female	38 (64.4)	30 (69.8)	8 (50)	0.5289
**Clinical manifestation**				
Proximal limbs weakness	57 (96.6)	42 (97.7)	15 (95.5)	0.4722
Distal limbs weakness	32 (54.2)	23 (53.5)	9 (56.3)	>0.9999
Muscle strength ≤3	32 (54.2)	25 (58.1)	7 (43.8)	0.3861
Cervical muscle weakness	6 (10.2)	4 (9.3)	2 (12.5)	0.6582
Dysphagia	6 (10.2)	5 (11.6)	1 (6.3)	>0.9999
Dyspnea	2 (3.4)	2 (4.7)	0	>0.9999
Muscle strength (MRC)	3.4 (2–5)	3.3 (2–5)	3.5 (3–5)	0.2681
Muscle atrophy	8 (13.6)	7 (16.3)	1 (6.3)	0.427
Myalgia	20 (33.9)	10 (23.3)	10 (62.5)	0.0114
Cardiac symptoms	1 (1.7)	1 (2.3)	0	>0.9999
EMA				
ILD	11 (18.6)	7 (16.3)	4 (25)	0.4681
Skin rash	4 (6.8)	4 (9.3)	0	0.5662
Arthritis	1 (1.7)	1 (2.3)	0	>0.9999
Raynaud phenomenon	0	0	0	>0.9999
**Examination**				
Initial CK, median (range), U/L	5,122.5 (25–20,000)	5,395 (25–17,100)	2,915 (414–20,000)	0.1075
Initial LDH, median (range), U/L	748 (143–2,712)	800 (143–2,712)	665.8 (226–1,705)	0.0856
Limb muscle MRI				
Edema	45 (78.9)	33 (78.6)	12 (80)	>0.7389
Fatty replacement	13 (22.8)	10 (23.8)	3 (20)	>0.9999
Atrophy	16 (28.1)	8 (19.0)	4 (26.7)	0.7132
Fascial edema	7 (12.3)	4 (9.5)	3 (20)	0.3645
Cardiac examinations				
ECG	14 (30.4)	8 (25)	6 (42.9)	0.3009
Echo	11 (22.9)	5 (15.2)	6 (46.2)	0.0509
Cardiac MRI	18 (0.6)	12 (50.0)	7 (100)	0.0261
**Pathological characteristics**	All patients (n = 55)	Seropositive (n = 39)	Seronegative (n = 16)	p-value
HE staining				
Necrotic fibers	55 (100)	39 (100)	16 (100)	>0.9999
Regeneration/degeneration	51 (92.7)	37 (94.9)	14 (87.5)	0.5713
Subtype of immune cells infiltration in IHC	All patients (n = 34)	Seropositive (n = 22)	Seronegative (n = 12)	
CD68^+^ macrophage	32 (94.1)	21 (95.4)	11 (91.7)	>0.9999
CD4^+^ T cells	25 (73.5)	15 (68.2)	10 (83.3)	0.4385
CD8^+^ T cells	27 (79.4)	17 (77.3)	10 (83.3)	>0.9999
CD20^+^ B cells	9 (26.5)	5 (22.7)	4 (33.3)	0.687
MHC-I upregulation	24 (70.6)	16 (72.7)	8 (66.7)	0.7139
MAC deposition on sarcolemma	19 (55.9)	15 (68.2)	2 (16.7)	0.0104
MAC deposition on blood vessels	25 (73.5)	14 (63.6)	11 (91.7)	0.1135
p62 expression on sarcoplasm	27 (79.4)	18 (81.8)	9 (75.0)	0.6769
**Treatment outcome**				
Onset to treatment, median (range), months	6 (0.5–48)	6 (0.5–48)	2 (0.5–48)	0.0144
Diagnosis to treatment, median (range), months	0 (0–3)	0 (0–3)	0 (0–2)	0.9251
Initial treatment				0.4522
Glucocorticoid monotherapy	34 (57.6)	23 (53.5)	11 (68.8)	
Glucocorticoid and Immunosuppressant	20 (33.9)	16 (37.2)	4 (25.0)	
Combined glucocorticoid with IVIg	2 (3.4)	1 (2.3)	1 (6.3)	
Glucocorticoid, Immunosuppressant and IVIg	3 (5.1)	3 (7.0)	0	
**Follow-up**				
Follow-up period, median (range), months	28 (5–132)	34 (5–132)	20 (12–84)	0.2491
Lost to follow-up	2 (3.4)	2 (4.7)	0	>0.9999
Medication at last follow-up				0.5872
None	10 (18.5)	6 (15.8)	4 (25.0)	
Glucocorticoid alone	6 (11.1)	4 (10.5)	2 (12.5)	
Immunosuppressant monotherapy	9 (16.7)	5 (13.2)	4 (25.0)	
Combined glucocorticoid with immunosuppressant	28 (51.9)	22 (57.9)	6 (37.5)	0.2358
Combined glucocorticoid with IVIg	1 (1.9)	1 (2.6)	0	
Outcome				
Death	3 (5.3)	3 (7.3)	0	
No improvement	0	0	0	
Mild improvement	5 (8.8)	5 (12.2)	0 (0)	
Moderate improvement	10 (16.9)	8 (19.5)	2 (12.5)	
Marked improvement	39 (66.1)	25 (61.0)	14 (87.5)	0.0641
Relapse	22 (38.6)	18 (43.9)	4 (25.0)	0.2357

*Data are n (%). p values comparing seropositive and seronegative groups are from χ^2^ test, Fisher's exact test, or Mann–Whitney U test. EMA, extra muscular disease activity; ILD, interstitial lung disease; MRC, Medical Research Council; CK, creatine kinase; ECG, electrocardiogram; Echo, echocardiogram; LDH, lactate dehydrogenase; MRI, magnetic resonance imaging; HE, hematoxylin–eosin; IHC, immunohistochemistry; MHC-I, major histocompatibility complex class I; MAC, membrane attack complex; IVIg, intravenous immunoglobulin*.

**Figure 2:**
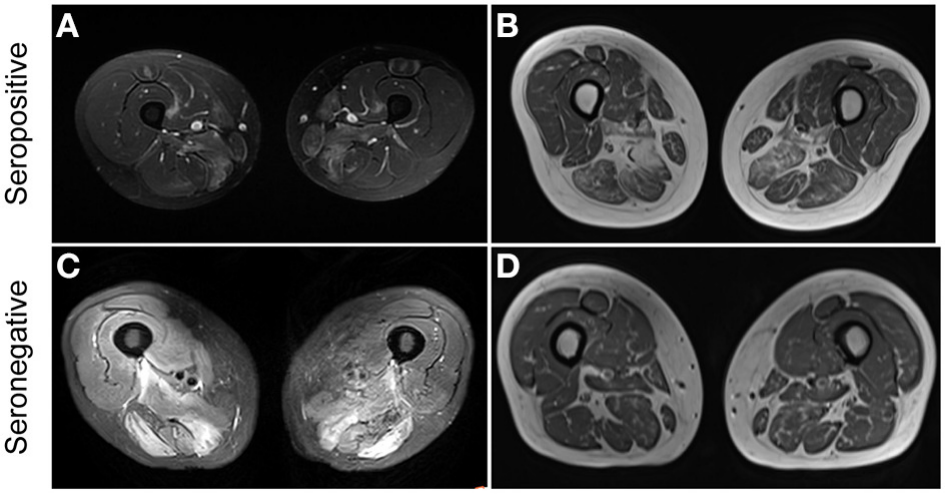
Representative thigh muscle MRI of seropositive and seronegative immune-mediated necrotizing myopathy (IMNM). **(A)** High signal intensity on MRI STIR images in a patient with seropositive IMNM. **(B)** Muscle atrophy and fatty replacement on MRI T1 images in a patient with seropositive IMNM. **(C)** Diffuse increased signals on MRI T2 images in a patient with seronegative IMNM. **(D)** Muscle atrophy and fatty replacement on MRI T1 images in a patient with seronegative IMNM.

Among patients with IMNM, seronegative patients tended to have lower serum LDH levels than individuals with seropositive IMNM [665.8 (226–1,705) vs. 800 (143–2,712), *p* = 0.0856]. Thigh muscle MRI with edema was frequent in both seropositive IMNM (78.6%) and seronegative IMNM (80%). Representative MRI images are displayed in Figure 2. Cardiac abnormalities (including systolic dysfunction, aortic stenosis, pericardial effusion, or enlargement of cardiac chambers detected by Echo and myocardial fibrosis, ventricular or atrial dilatation, or myocardial ischemia detected by cardiac MRI) were frequently observed in patients with seronegative IMNM (Echo, 6/13 vs. 5/33, *p* = 0.0509; cardiac MRI, 7/7 vs. 12/24, *p* = 0.0261).

**Figure 3:**
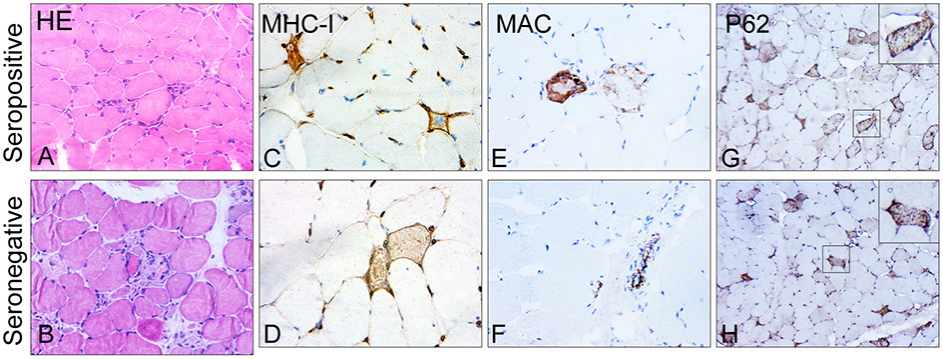
Representative images of major histocompatibility complex class I (MHC-I), membrane attack complex (MAC), and p62 in skeletal muscle biopsy of patients with seropositive and seronegative immune-mediated necrotizing myopathy (IMNM). **(A,B)** Representative images of hematoxylin–eosin (HE)-stained sections showing numerous necrotic and regenerating fibers. Original magnification: ×200. **(C,D)** Representative images of upregulation of MHC-I. Original magnification: ×400. **(E,F)** Representative images of sarcoplasmic MAC deposition on fibers in biopsied samples of patients with seropositive IMNM and vascular immunostaining pattern for MAC in patients with seronegative IMNM. Original magnification: ×400. **(G,H)** Representative images of a diffusely fine and homogeneous staining pattern with variable intensity of P62 in muscle biopsies. Original magnification: ×200.

In the semi-quantitative analysis of inflammatory infiltrates, we observed no differences in the presence of CD68^+^ macrophages, CD4^+^ T lymphocytes, CD8^+^ T lymphocytes, or CD20^+^ B lymphocytes between the two groups (Supplementary Figures 2A,B). Deposition of MAC on the sarcolemma of necrotic or non-necrotic myofibers was more frequently observed in patients with seropositive IMNM than in those with seronegative IMNM (68.2 vs. 16.7%, *p* = 0.0104). There were no significant differences in the degree of MHC-I upregulation on myofibers, deposition of MAC on vessels, or sarcoplasmic staining of p62 between the two groups (Figure 3).

The interval between the onset of symptoms and initiation of immunotherapy was shorter in seronegative patients than that in seropositive patients (*p* = 0.0144). By the last interview, three patients with anti-SRP antibodies had died of severe pulmonary infections and respiratory failure, and lung cancer occurred in an anti-SRP antibody-positive patient 1 year after the diagnosis of IMNM. The rate of marked improvement tended to be higher in patients with seronegative IMNM than in those with seropositive IMNM (87.5 vs. 61.0%, *p* = 0.0641). There were no significant differences in the interval from diagnosis to initial treatment, follow-up period, medication at last follow-up, outcome distribution, or the rate of recurrence between seropositive and seronegative patients.

**Figure 4:**
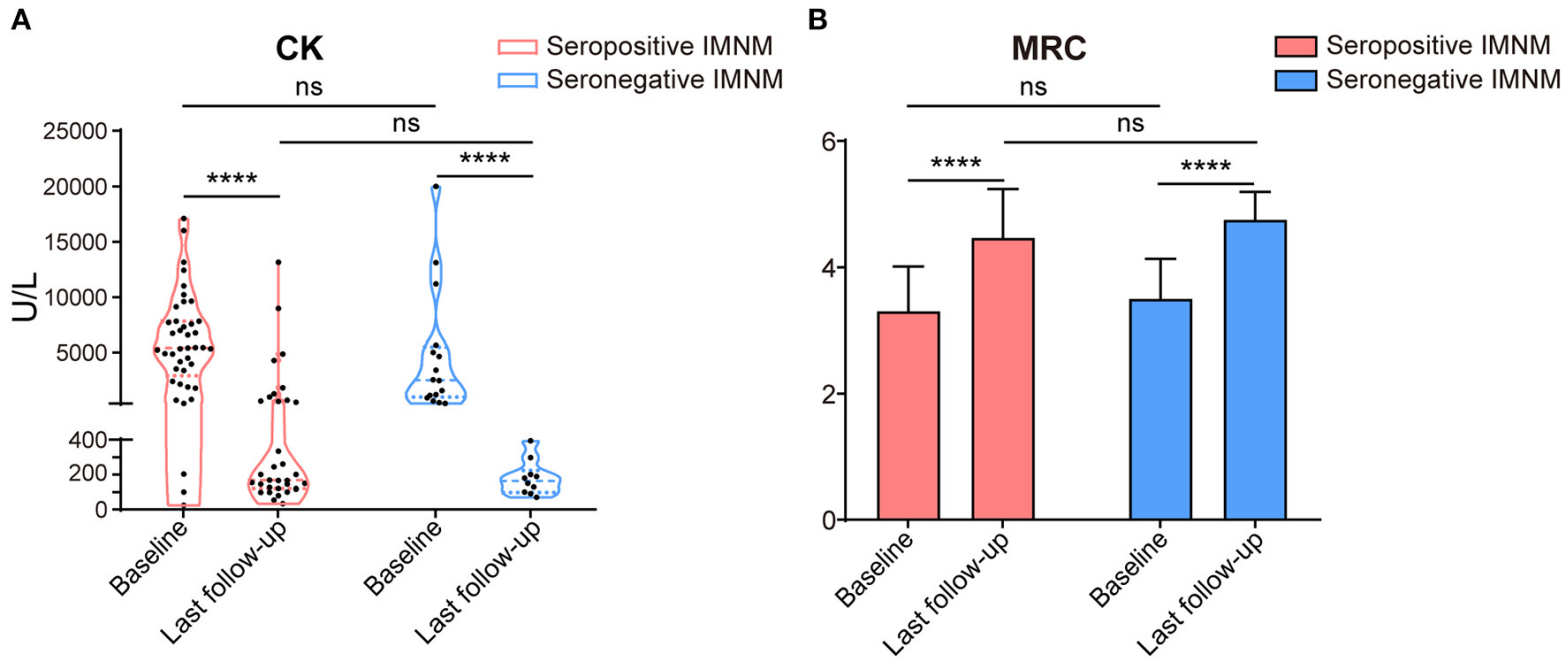
Changes of serum creatine kinase levels and Medical Research Council (MRC) at base and by the last follow-up of patients with seropositive and seronegative immune-mediated necrotizing myopathy (IMNM). **(A)** A violin plot with median and interquartile range of serum creatine kinase (CK) levels of the seropositive and seronegative patients at baseline and last follow-up (for seropositive IMNM group: baseline *n* = 41, last follow-up *n* = 33; for seronegative IMNM group: baseline *n* = 16, last interview *n* = 10). ****p < 0.0001; ns, no significance. **(B)** A histogram with median and standard deviation of MRC grade of the patients with seropositive IMNM and seronegative IMNM (for seropositive IMNM group: baseline *n* = 43, last follow-up *n* = 41; for seronegative IMNM group: baseline *n* = 16, last interview *n* = 10). ****p < 0.0001; ns, no significance.

Changes in the serum CK levels and MRC grades of patients with seropositive and seronegative IMNM at baseline and at the last follow-up are illustrated in Figures 4A,B, respectively. Overall, serum CK levels significantly decreased to the normal range (*p* < 0.0001), and the MRC scores significantly improved (*p* < 0.0001) by the last follow-up in both seropositive and seronegative IMNM groups. However, there were no differences in serum CK levels or MRC grades between seropositive and seronegative patients at onset or at the last follow-up.

## Discussion

In this study, we compared detailed clinicopathological characteristics, treatment strategies, and outcome data between Chinese patients with seronegative IMNM and those with anti-SRP antibody-positive IMNM or anti-HMGCR antibody-positive IMNM (i.e., termed seropositive IMNM). Our data indicated that seronegative IMNM frequently presented with myalgia and subclinical cardiac abnormalities, while MAC deposition on the sarcolemma was uncommon relative to that observed in seropositive IMNM. Furthermore, patients with seronegative IMNM experienced better outcomes than those with seropositive IMNM.

To date, few reports have discussed seronegative IMNM. In previous European studies, the proportion of patients with MSA-negative IMNM or both anti-SRP and anti-HMGCR antibody-negative IMNM was 21.4–40% (4, 8, 25, 26). So far, the prevalence of seronegative IMNM in China has been unknown. Our data revealed that seronegative IMNM accounted for 13.7% of IMNM cases in our center. Unlike previous studies on MSA-negative MNM (8, 13), we did not observe female predominance or higher rates of cancer among seronegative patients. The lower percentage of seronegative patients with IMNM in the current study and these differences in characteristics from MSA-negative IMNM were presumably due to the more stringent inclusion criteria in the present study. Specifically, MSA-positive IMNM, cancer-associated IMNM, and CTD-related IMNM were no longer the etiological targets of our investigation.

Cardiac abnormalities were frequently detected in seronegative IMNM, although no patient reported any cardiac symptoms at presentation. Previous reports on cardiac involvement in autoimmune myopathies have indicated that cardiac involvement more frequently occurred in patients with anti-SRP antibody-positive IMNM (3) likely because such studies neglected patients with seronegative IMNM. In the current study, cardiac ultrasound and cardiac MRI (but not ECG) frequently detected cardiac involvement in patients with seronegative IMNM. ECG is ordinarily used to detect cardiac abnormalities but exhibits low sensitivity. Consistent with the findings of previous studies (27, 28), our results also suggested that cardiac MRI is more sensitive for detecting heart abnormalities in patients with IMNM. The more frequent cardiac involvement in seronegative IMNM suggests a complex mechanism of cardiac tissue damage in these patients. Longitudinal studies concerning whether early and augmented therapeutic strategies targeting subclinical cardiac involvement will be beneficial to the outcome of seronegative IMNM are therefore required.

Sarcolemmal MAC deposition tended to be more frequent among patients with seropositive IMNM than among those with seronegative IMNM, indicating that humoral immunity may not play a key role in the pathophysiology of seronegative IMNM. Nevertheless, the potential role of autoimmunity in seronegative IMNM should be explored.

Most patients with seronegative IMNM on formal immunotherapy achieved satisfactory recovery. In general, seronegative IMNM subsequently required adjuvant immunosuppressants to achieve sufficient recovery, to reduce the dose of glucocorticoid treatment, or to prevent relapse, even though most patients responded favorably to high-dose glucocorticoid alone at onset. These findings are in accordance with those of previous studies on seropositive IMNM (3, 25, 29). A previous study reported that 38% of patients with MSA-negative IMNM had good to excellent outcomes and that 47% had moderate outcomes (8). In the current study, marked improvement was observed in 87.5% of patients with seronegative IMNM, a rate higher than that observed in seropositive patients. The better prognosis among seronegative IMNM may be associated with the shorter interval between the onset of myopathy and the initiation of immunotherapy in seronegative patients or other unknown reasons. The symptoms of weakness and myalgia are largely attributed to persistent and active immune-mediated muscle damage. Previous research regarding the treatment of IMNM concluded that early treatment initiation can help to achieve early remission (3, 30), which to a certain degree supports our speculation. Therefore, early initiation of immunotherapy may be of great necessity.

In addition, 25% of seronegative patients in our study experienced a relapse while reducing the dose of prednisone, following which they slowly recovered after the introduction of immunosuppressive treatment. Therefore, long-term follow-up and regular monitoring of disease activity are necessary, and aggressive immunotherapy should be considered if seronegative patients exhibit signs of recurrence. This suggestion has also been offered in other reports on IMNM (3, 29, 31). Clinical parameters such as MRC score, CK levels, muscle MRI findings, and even evaluations of ILD and cardiac abnormalities are currently the key indices for monitoring disease activity during long-term follow-up. More detailed clinical trials are needed to identify reliable markers for monitoring disease activity.

The present study had some limitations, including its limited sample size and single-center retrospective design. Therefore, information scarcity and selection bias may exist. A prospective study with a larger sample of patients treated across multiple medical centers will further contribute to clarify the features of patients with seronegative IMNM. Otherwise, line blotting was used to detect MSAs and MAAs in patients with myositis in our study. The percentage of false negatives and false positives generated by these two commercial assays is of great concern (32, 33). To eliminate or minimize this issue, each included participant was tested for MSAs and MAAs at least twice.

In conclusion, our findings demonstrated that attempting immunotherapy after excluding hereditary myopathy and muscular dystrophy may be helpful when patients with necrotizing myopathies present with certain symptoms. Even without reliable clues indicative of immune disorders, this strategy may be effective in patients exhibiting myalgia at presentation, subclinical cardiac abnormalities, and uncommon sarcolemmal MAC deposition in skeletal muscle.

## Data Availability Statement

The raw data supporting the conclusions of this article will be made available by the authors, without undue reservation.

## Ethics Statement

The studies involving human participants were reviewed and approved by The Institutional Review Board at Tongji Hospital of Tongji Medical College, Huazhong University of Science and Technology. Written informed consent to participate in this study was provided by the participants' legal guardian/next of kin. Written informed consent was obtained from the individual(s), and minor(s)' legal guardian/next of kin, for the publication of any potentially identifiable images or data included in this article.

## Author Contributions

BB has full access to all the data in the study and takes responsibility for the integrity of the data and the accuracy of the data analysis, contributed to critical revision of the manuscript for important intellectual content, and contributed to the supervision. XM and BB contributed to the concept and design. XM, LX, SJ, YL, and BB contributed to the acquisition, analysis, and interpretation of data. XM contributed to drafting of the manuscript. XM, XL, SJ, and YL contributed to the statistical analysis. XM and XL contributed to the administrative, technical, and material support. All authors contributed to the article and approved the submitted version.

## Conflict of Interest

The authors declare that the research was conducted in the absence of any commercial or financial relationships that could be construed as a potential conflict of interest.
