# Supplementary material for: The Clinicopathological Distinction Between Seropositive and Seronegative Immune-Mediated Necrotizing Myopathy in China
Source: Front Neurol. 2021 Jul 5;12:670784. doi: 10.3389/fneur.2021.670784 (PMC8287052; doi:10.3389/fneur.2021.670784)
Supplement: Supplementary file 1 [file Data_Sheet_1.docx]

Supplementary Material

# Supplementary Figures and Tables

## Supplementary Figures


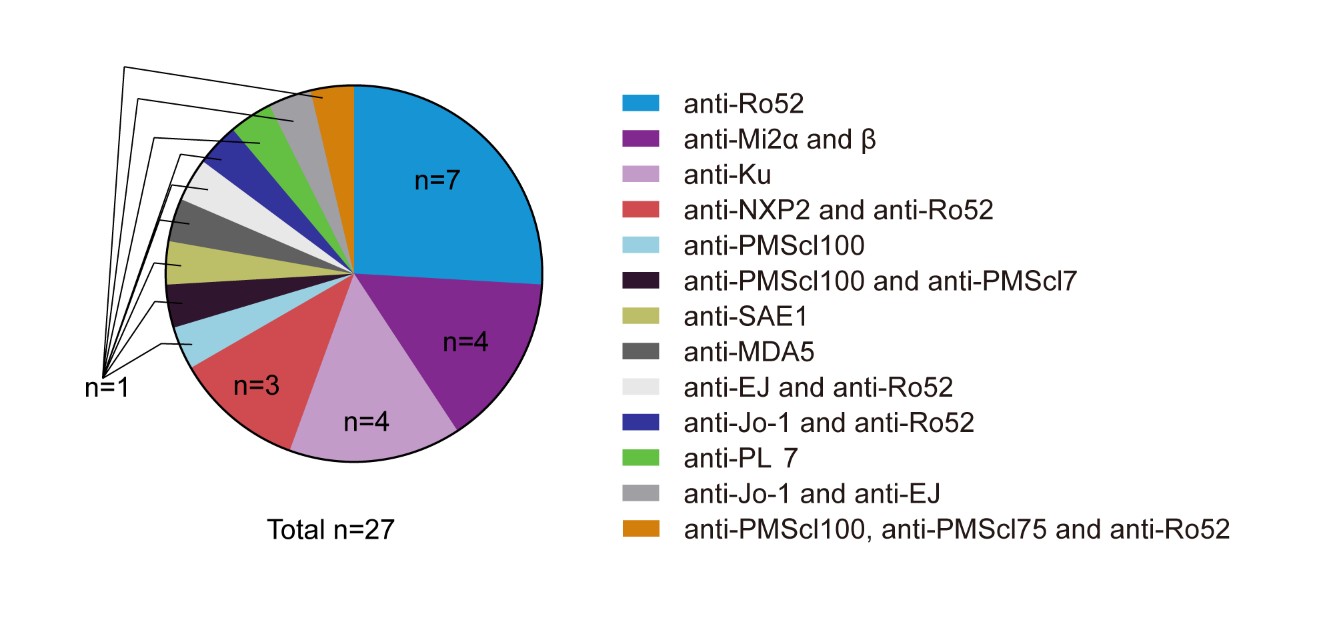


**Supplementary Figure 1.The distribution of excluded IMNM patients with other MSAs or MAAs.**


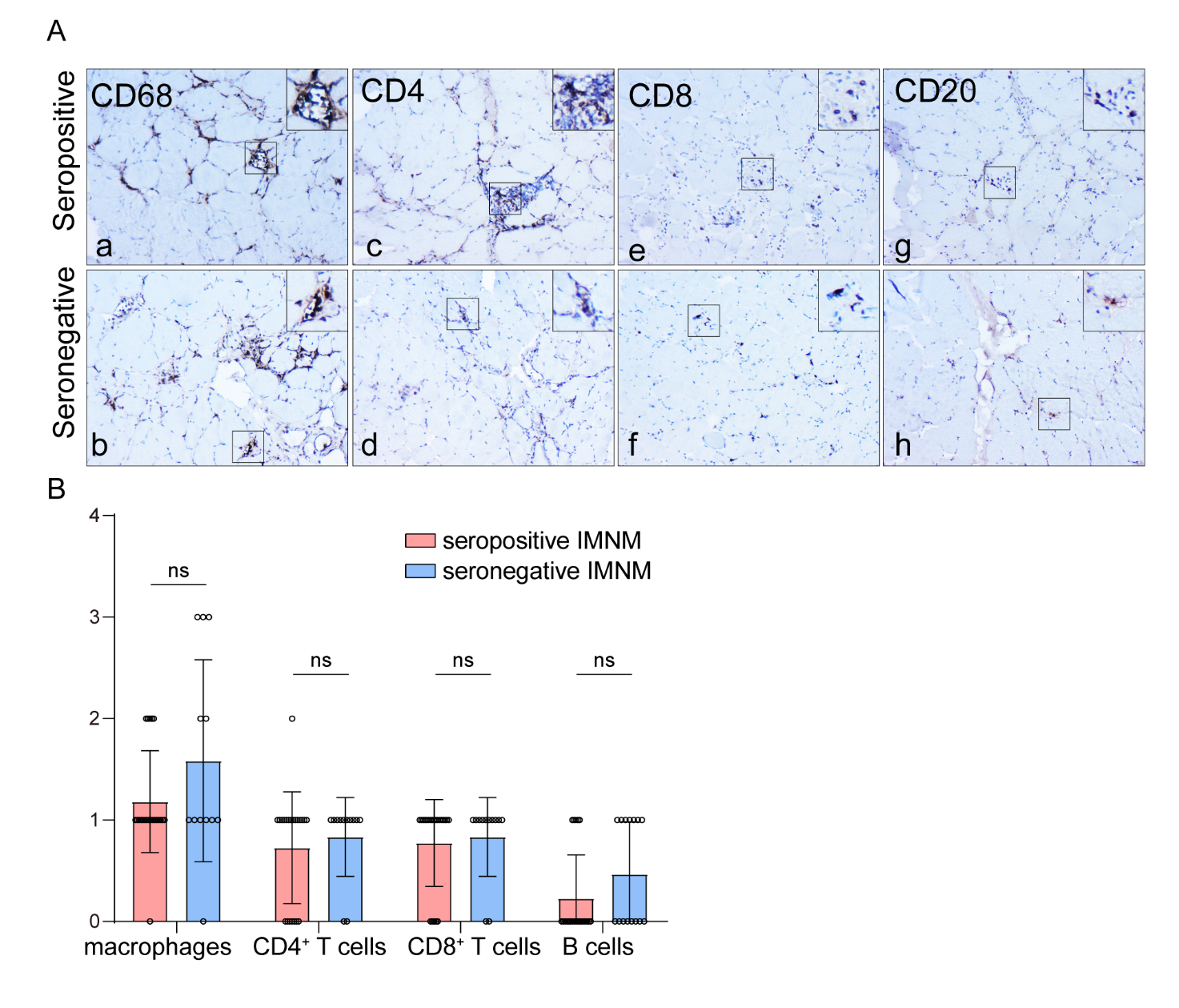


**Supplementary Figure 2.Characterization of inflammatory cells in biopsied samples of seropositive and seronegative IMNM.**

Aa and Ab: Representative images of scattered or focal CD68^+^ macrophages in biopsied specimens of seropositive IMNM and seronegative IMNM. Original magnification: X200.

Ac and Ad: Representative images of scattered CD4^+^ T lymphocytes. Original magnification: X200.

Ae and Af: Scattered CD8^+^ T lymphocytes were detected in biopsied samples. Original magnification: X200.

Ag and Ah: Representative images of a few CD20^+^ B cells in skeletal muscle. Original magnification: X200.

B: Semi-quantitative analysis of CD68^+^ macrophages, CD4^+^ T lymphocytes, CD8^+^ T lymphocytes and CD20^+^ B cells was shown in the dot-plot with median and standard deviation. ns, no significance.

**1.2 Supplementary Table 1 Clinical characteristics of 16 patients with seronegative IMNM**

| Patient No. | Onset age, y/sex | Onset to treatment, months | No. ofrelapse | Clinical presentation | CK at onset | Treatment | Follow-up period, month | Outcome | Cardiac involvement |
| --- | --- | --- | --- | --- | --- | --- | --- | --- | --- |
| 1 | 24/F | 2 | 1 | Cervical muscle weakness, proximal and distal weakness, dysphasia, myalgia | 4642 | CS, MMF | 19 | Marked improvement | NA |
| 2 | 33/M | 0.5 | 0 | Proximal weakness, myalgia | 2471 | CS, FK506 | 28 | Marked improvement | Left atrial enlargement |
| 3 | 36/M | 0.5 | 0 | Myalgia, ILD | 20000 | CS | 32 | Marked improvement | Focal fibrosis, atrial dilatation, and pericardial effusion |
| 4 | 40/F | 1 | 0 | Proximal weakness | 3435 | CS, MTX | 47 | Marked improvement | Left ventricle hypertrophy and focal fibrosis |
| 5 | 45/M | 0.5 | 0 | Proximal and distal weakness, ILD | 925 | CS, FK506 | 15 | Marked improvement | NA |
| 6 | 48/F | 4 | 1 | Proximal and distal weakness | 485 | CS, FK506 | 28 | Marked improvement | NA |
| 7 | 62/M | 6 | 0 | Severe proximal and distal weakness, myalgia | 414 | CS, FK506 | 23 | Marked improvement | Left ventricular enlargement and focal fibrosis |
| 8 | 63/M | 2 | 0 | Sever proximal weakness, myalgia | 642 | CS | 15 | Moderate improvement | Atrioventricular block, aortic stenosis of double-valve pattern |
| 9 | 66/M | 1 | 0 | Sever proximal weakness, ILD, myalgia | 5658 | CS | 12 | Marked improvement | NA |
| 10 | 69/M | 12 | 0 | Severe proximal and distal weakness | 1591 | CS, IVIg | 12 | Marked improvement | Left anterior fascicular block, enlarged and dysfunctional systolic left ventricular |
| 11 | 53/F | 7 | 0 | Proximal and distal weakness, myalgia | 1230 | CS | 20 | Marked improvement | Abnormal repolarization of myocardium |
| 12 | 43/F | 2 | 1 | Cervical muscle weakness, proximal weakness, myalgia | 1156 | CS, FK506 | 14 | Marked improvement | Sinus tachycardia and focal fibrosis |
| 13 | 42/M | 0.5 | 0 | Severe proximal and distal weakness | 11192 | CS | 48 | Marked improvement | Focal fibrosis and pericardial effusion |
| 14 | 48/F | 48 | 1 | Proximal weakness | 2567 | CS, AZA | 77 | Moderate improvement | Frequent atrial premature with intraventricular aberrant conductions, myocardial ischemic, left ventricular remodeling and pericardial effusion |
| 15 | 73/F | 3 | 0 | Severe proximal and distal weakness, myalgia, ILD | 13116 | CS, FK506 | 18 | Marked improvement | Changes of T wave and lateral wall leads, ischemic changes and focal fibrosis |
| 16 | 44/F | 9 | 0 | Proximal and distal weakness, myalgia | 5000 | CS, MTX | 84 | Marked improvement | NA |

Abbreviations: AZA, azathioprine; CK, creatinine kinase; CS, glucocorticoids; F, female; FK506, tacrolimus; ILD, interstitial lung disease; M, male; MTX, methotrexate; MMF, mycophenolate mofetil; NA, not applicable; No., number; RTX, rituximab; +, presence; -, none
